# Foodborne Transmission of Bovine Spongiform Encephalopathy to Nonhuman Primates

**DOI:** 10.3201/eid1905.120274

**Published:** 2013-05

**Authors:** Edgar Holznagel, Barbara Yutzy, Walter Schulz-Schaeffer, Carina Kruip, Uwe Hahmann, Pär Bierke, Juan-Maria Torres, Yong-Sun Kim, Achim Thomzig, Michael Beekes, Gerhard Hunsmann, Johannes Loewer

**Affiliations:** Paul-Ehrlich-Institut, Langen, Germany (E. Holznagel, B. Yutzy, C. Kruip, J. Loewer);; University of Göttingen, Göttingen, Germany (W. Schulz-Schaeffer);; German Primate Centre, Göttingen (U. Hahmann, G. Hunsmann);; Swedish Institute for Infectious Disease Control, Solna, Sweden (P. Bierke);; Centro de Investigación en Sanidad Animal, Madrid, Spain (J.-M. Torres);; Hallym University, Anyang, Gyeonggi-Do, South Korea (Y.-S. Kim); Robert-Koch-Institut, Berlin, Germany (A. Thomzig, M. Beekes)

**Keywords:** BSE, bovine spongiform encephalopathy, Creutzfeldt-Jakob disease, dietary, foodborne, incubation period, *Macaca fascicularis*, prions and related diseases, nonhuman primates

## Abstract

Risk for human exposure to bovine spongiform encephalopathy (BSE)–inducing agent was estimated in a nonhuman primate model. To determine attack rates, incubation times, and molecular signatures, we orally exposed 18 macaques to 1 high dose of brain material from cattle with BSE. Several macaques were euthanized at regular intervals starting at 1 year postinoculation, and others were observed until clinical signs developed. Among those who received ≥5 g BSE-inducing agent, attack rates were 100% and prions could be detected in peripheral tissues from 1 year postinoculation onward. The overall median incubation time was 4.6 years (3.7–5.3). However, for 3 macaques orally exposed on multiple occasions, incubation periods were at least 7–10 years. Before clinical signs were noted, we detected a non-type 2B signature, indicating the existence of atypical prion protein during the incubation period. This finding could affect diagnosis of variant Creutzfeldt-Jakob disease in humans and might be relevant for retrospective studies of positive tonsillectomy or appendectomy specimens because time of infection is unknown.

Variant Creutzfeldt-Jakob disease (vCJD) (*1*) is most likely caused by dietary exposure to bovine spongiform encephalopathy (BSE) prions (*2*–*4*). In the United Kingdom, risk for infection with BSE has been considerable, but only 172 cases of vCJD have been documented (*5**–**7*). However, the infective dose for oral transmission of the BSE agent to humans is unknown, and incubation times can only be estimated (*5*,*6*). In 2001, the European Union funded a risk assessment study in nonhuman primates to estimate the risk for humans exposed to BSE-contaminated food or blood products (*8*).

A determining factor for susceptibility to BSE prions is a polymorphism for methionine (M) or valine (V) at codon 129 of the human prion protein gene (*PRNP*). All vCJD cases examined were methionine homozygotes at *PRNP* codon 129 (129-MM) (*9*). The overall distribution of *PRNP* codon 129 genotypes in the general UK population is ≈39% MM, ≈50% MV, and ≈11% VV (*7*,*10*). Evidently, persons with a 129-VV genotype can be infected (*11*), and clinical signs develop after a longer incubation time among those with a 129-MV genotype than among those with a 129-MM genotype (*12*). However, retrospective analyses of biopsy samples suggest that prevalence of BSE infection is higher among persons who belong to a certain birth cohort and lived in the United Kingdom from 1980 through 1989 (109 cases/million persons [*13*] to 237 cases/million persons [*14*]). The reason for the discrepancy between the low number of vCJD cases and higher prevalence of infected persons in the United Kingdom is not known, but the *PRNP* polymorphism might contribute to this discrepancy as just described. Intriguingly, among hamsters that were orally exposed multiple times to central nervous system (CNS) tissues infected with the scrapie agent, incubation times were significantly prolonged (*13*). Thus, not only the *PRNP* polymorphism and the dose but also the mode of transmission might contribute to the development of subclinical cases. However, estimating exposure risks for humans based solely on these results is difficult because of the digestive physiology, life expectancy, and other metabolic parameters of hamsters.

In prion diseases such as CJD, kuru, BSE, scrapie, and chronic wasting disease, the cellular form of prion protein (PrP^C^) is thought to be converted into abnormal PrP (PrP^Sc^) through a posttranslational event. As a result, PrP^Sc^ becomes partially resistant to proteases. The misfolded prion protein comprises an N terminal protease-sensitive part followed by a region with variable protease sensitivity and a C-terminal protease-resistant core referred to as PrP^res^ or PrP27–30 (Figure 1). Limited protease exposure of PrP^Sc^ in vitro generates nonglycosylated core fragments of 19–21 kDa (*14*,*15*), which are used to distinguish 2 major PrP^Sc^ types by electrophoresis. Type 1 core protein has an apparent molecular mass of 21 kDa. Its primary cleavage site is at residue 82. Type 2 core protein migrates to the 19-kDa region and has a primary cleavage site at residue 97 (*16*,*17*). Both types can coexist in a considerable number of sporadic CJD (*18*,*19*) and vCJD cases (*14*). Subtypes and strains can be further characterized by their so-called glycoform profile because the nonobligatory addition of 1–2 sugar chains results in 3 differently glycosylated isoforms in PrP^C^ and PrP^Sc^ (non-, mono-, and diglycosylated molecules, referred to as a PrP^res^ triplet). In sporadic CJD type 2, the monoglycosylated isotype predominates and is referred to as a type 2A signature; whereas, in vCJD, the diglycosylated isoform predominates (*1*) and is referred to as a type 2B signature (*14*,*16*,*19*). Additional PrP^res^ fragments have been described, for example the so-called C-terminal fragments of 12/13 kDa (*20*) and 17 kD (*21*).

**Figure 1:**
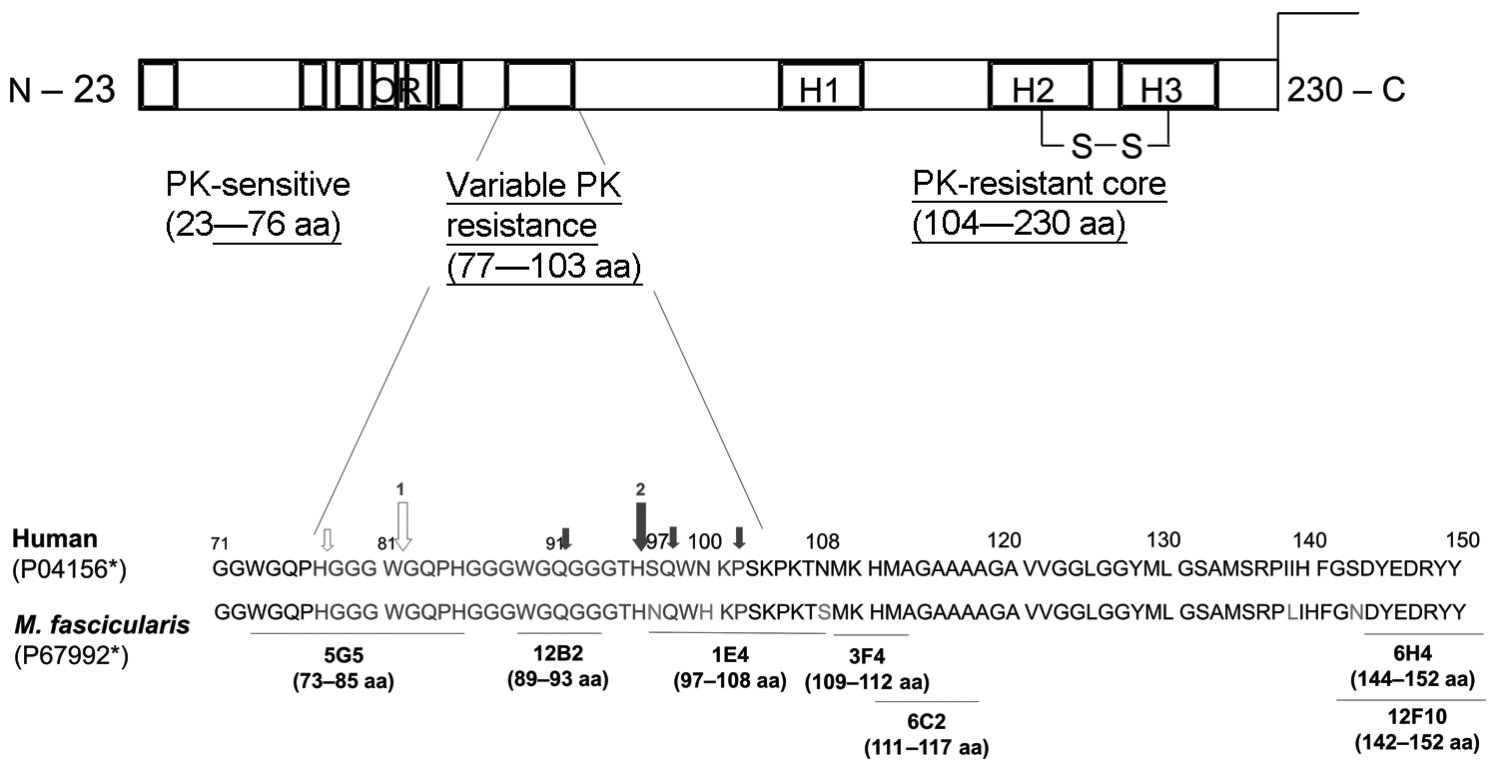
Schematic diagram of the mature nonglycosylated prion protein and below amino acid sequences of the human and the simian prion polypeptide chain. Homology (198/207 aa) between human and simian mature cellular form of prion protein on the amino acid level is 96%. Large and small arrows indicate major and minor, respectively, proteinase K digestion sites (*14*). Open arrows indicate digestion sites in type 1 fragments; filled arrows indicate digestion sites in type 2 fragments. The epitopes recognized by the used monoclonal antibodies are shown below. OR, octarepeat region; H, α-helical structure; PK, proteinase K; *M. fascicularis,* *Macaca fascicularis.* *Swiss-Prot (www.ebi.ac.uk/swissprot/*)* accession numbers.

Results of the European Union–funded nonhuman primate risk assessment study, designed to determine the dose at which 50% of macaques will be infected (*8*), show that a 5-g dose given on 1 occasion infected all macaques. Moreover, multiple exposures to high doses might prolong incubation time. Intriguingly, a non–type 2B PrP^res^ pattern in CNS tissues of macaques during the preclinical phase indicated the existence of an intermediate prion isoform. This finding might be relevant for retrospective studies of tonsillectomy or appendectomy specimens, because the time point of infection in humans with PrP^res^-positive biopsy specimens is not known. As part of the European Union–funded study, we aimed to determine attack rates and incubation times after oral exposure to 5 g or 16 g of BSE-infected brain material in adult cynomolgus monkeys (*Macaca fascicularis*).

## Methods

We orally exposed 18 macaques, each 5 years of age, to brain material from cattle with BSE: 15 macaques on 1 occasion and 3 macaques on multiple occasions. Most animals were kept at the primate center of the Paul-Ehrlich-Institut under biosafety level 3 conditions; the others (macaques M3–M8) were kept at the Swedish Institute for Infectious Disease Control, Stockholm. The study was approved by the Hessian Animal Protection Committee (local permit no. F107/45 and F107/63) and supervised by local authorities (Regierungspräsidium Darmstadt).

The BSE inoculum was a pool of homogenized bovine brain from 11 cows with natural BSE infection (*22*). The level of infectivity was determined (data not shown) in BoTg110 mice (*23*). Cynomolgus monkeys were purchased from the Centre de Recherche en Primatologie, Mauritius. All animals were homozygous for M at codon 129 of the *PRNP* gene (*4*,*22*). We fed (in muesli balls, monitored by video to ensure that the total amount was eaten) 5 g of BSE inoculum to each of 12 macaques (macaques S1–S5 and S9–S15), 8–16 g of BSE inoculum to each of 6 macaques (macaques S6–S8 and C1–C3), and mock inoculum (non-BSE–infected brain material) to each of 8 macaques (macaques M1–M8). We had originally planned to inoculate all macaques on 1 occasion; however, because of feeding problems, 3 macaques (C1–C3) had to be inoculated on several occasions. Of these 3 macaques, 1 received a cumulative dose of 8 g, the second 10 g, and the third 16 g (Table 1).

**Table 1:** Schedule for successive oral inoculation of 3 macaques with brain tissue from BSE-infected cattle*

Macaque	Dose, g				
	Day 1	Day 2	Day 3	Day 7	Cumulative
C1	5.0	2.0	None	1.0	8.0
C2	6.4	2.6	None	1.0	10.0
C3	9.6	3.5	2.9	None	16.0
*BSE, bovine spongiform encephalopathy.					

All animals were observed daily for any abnormalities. Cerebrospinal fluid (CSF) samples were collected at regular intervals and examined for biomarkers of brain damage by 14–3-3 protein (14–3-3p) tests (*22*). We planned 2 studies. For study 1, BSE-infected macaques (groups I and III) were to be kept until development of clinical signs to determine incubation periods. For study 2, macaques (group II) received 5 g BSE brain material on 1 occasion and were euthanized at regular intervals during the incubation period (macaques S9–S15, Table 2). However, study 1 was possible only for macaques S1–S8 (group I, Table 2) because among macaques C1–C3 (group III, Table 2), non–BSE-associated disease necessitated euthanasia. Macaques M1–M8 were the control macaques (group IV) inoculated with non-BSE brain material (Table 2).

**Table 2:** Characteristics of oral inoculation of 5-year-old macaques with BSE-inducing or mock brain material *

Macaque	Sex	BSE dose, g	Euthanized, y postinoculation	14-3-3p–positive CSF	PrP^res^ pattern (brain)	PrP^res^ pattern (spinal cord C_1_–T_12_)	PrP^res^ pattern (spinal cord L_1_–L_4_)
Group I (clinically infected)†							
S1	F	5	4.3	Yes	2B	2B	2B
S2	F	5	4.6	Yes	2B	2B	2B
S3	M	5	4.7	Yes	2B	2B	2B
S4	F	5	4.8	Yes	2B	2B	2B
S5	F	5	5.2	Yes	2B	2B	2B
S6‡	F	16	3.7	Yes	2B	2B	2B
S7	F	16	4.5	Yes	2B	2B	2B
S8	F	16	5.3	Yes	2B	2B	2B
Group II (preclinical)§							
S9	M	5	1.0	No	Neg	Neg	Neg
S10	M	5	1.0	No	Neg	Neg	Neg
S11	M	5	3.0	No	Neg	Neg	Non-2B
S12	M	5	3.0	No	Neg	Neg	Non-2B
S13	M	5	3,9	No	Neg	Th_7–10_+	Neg
S14	F	5	4,1	No	Neg	Neg	Non-2B
S15	F	5	5.0	No	Neg	Neg	Non-2B
Group III (preclinical)¶							
C1	F	8	6.5	No	Neg	Neg	Non-2B
C2	F	10	6.5	No	Neg	Neg	Non-2B
C3	F	16	8.8	No	Neg	Neg	Neg
Group IV (controls)#							
M1	F	5	2.0	No	Neg	Neg	Neg
M2	F	5	5.0	No	Neg	Neg	Neg
M3	F	16	6.0	No	Neg	Neg	Neg
M4	F	16	6.0	No	Neg	Neg	Neg
M5	F	16	6.0	No	Neg	Neg	Neg
M6	F	16	6.0	No	Neg	Neg	Neg
M7	F	0.05	6.0	No	Neg	Neg	Neg
M8	F	0.05	6.0	No	Neg	Neg	Neg

*BSE, bovine spongiform encephalopathy; PrP^res^, proteinase-resistant prion protein; CSF, cerebrospinal fluid; C, cervical; T, thoracic; L, lumbar; neg, negative. †Received 1 BSE dose, observed until onset of clinical signs. ‡Macaque S6 had a highly stimulated immune system on the day of oral exposure and thereafter (reason unknown) and had the highest postmortem levels of proteinease-resistant prion protein (PrP^res^) in spleen and other lymphoreticular tissues of all examined macaques (data not shown). §Animals received 1 BSE dose and were euthanized at regular intervals during incubation period (all animals had PrP^res^ -positive non–central nervous system tissue). ¶Cumulative BSE dose, euthanized. #Exposed to mock (non–BSE-infected) bovine brain material and euthanized during aging to act as age-matched controls.

During postmortem examinations, brain, spinal cord, gut-associated lymphoid tissues (GALT), lymph nodes, tonsils, and other organs and tissues were either fixed in buffered formaldehyde solution (4% wt/vol) or stored at −80°C as described (*22*). Routine histopathologic examinations of the brains were performed to detect spongiform lesions in hematoxylin and eosin–stained tissue sections. To detect proteinase K (PK)–resistant PrP fragments in tissue sections, we conducted paraffin-embedded tissue blot analyses (*24*). Western immunoblot analyses to localize PrP^res^ in homogenized and PK–treated (20 µg PK/mL buffer) tissue samples (50 μg of tissue proteins were loaded onto a lane) were conducted as described (*22*). Western blot–negative results were retested by using Amersham Hyperfilm ECL (GE Healthcare, Life Sciences Europe, Freiburg, Germany) for visualization. Monoclonal antibodies and polyclonal antiserum were used for immunodetection (Table 3) and epitope mapping of PrP^C^ and PrP^Sc^ (Figure 1). Bioassay studies were conducted in BoTg110 mice expressing the bovine *PRNP* gene (*23*).

**Table 3:** Products used for Western immunoblot and paraffin-embedded tissue blot analyses*

mAb or antiserum	Linear epitope (amino acid) on human PrP	Source (reference)
mAb 8B4	37–44	Santa Cruz Biotechnology, Santa Cruz, CA, USA
mAb 5G5	73–85	(*25*)
mAb 12B2	89 −93	CIDC, Lelystad, the Netherlands (*14*)
mAb 1E4	97–108	Sanquin BV, Amsterdam, the Netherlands (*14*)
mAb 3F4	109–112	Abcam, Cambridge, UK
mAb F89-160-1-5	142–152	Calbiochem/Merck4Biosciences, Darmstadt, Germany
mAb 1C5	119–130	(*26*)
mAb 6H4	144–152	Prionics AG, Schlieren, Switzerland
mAb 12F10	142–152	SPI-Bio/IBL Int., Hamburg, Germany
C-20 antiserum	220–231	Santa Cruz Biotechnology, Santa Cruz, CA, USA
mAb BAR 236	213– 251	SPI-Bio/IBL Int., Hamburg, Germany
*mAb, monoclonal antibody; PrP, prion protein.		

## Results

**Figure 2:**
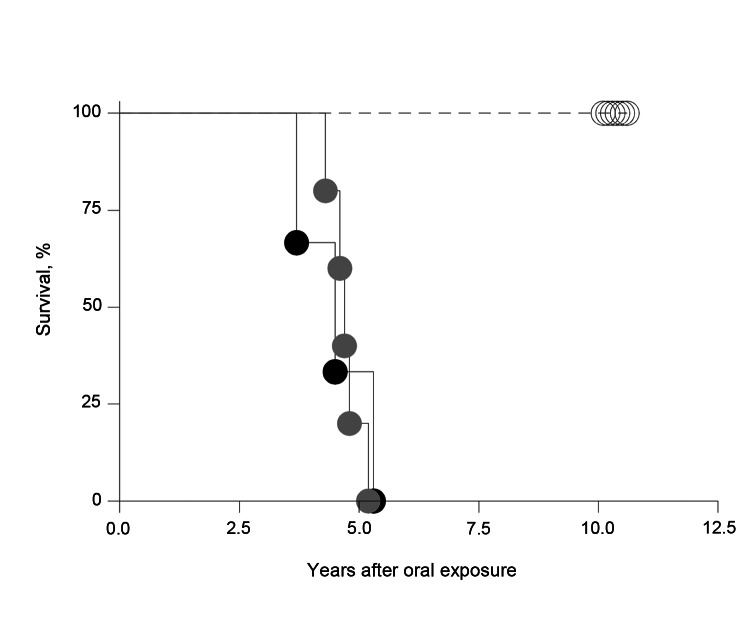
Percentage macaques surviving after oral inoculation brain material with or without (mock) bovine spongiform encephalopathy (BSE)–-inducing agent. Macaques exposed to 5 g (gray circles) or 16 g BSE (black circles) on 1 occasion and mock controls (open circles) are shown. The median incubation times for those given 16 g and 5 g BSE each was 4.7 years and 4.6 years, respectively. The difference was statistically not significant.

**Figure 3:**
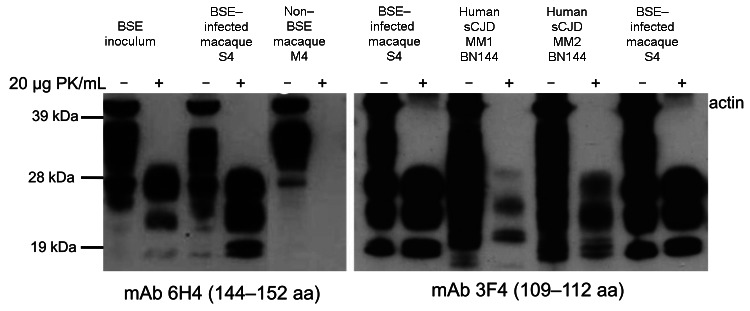
Western immunoblot analysis of proteinease-resistant prion protein (PrP^res^) (from brain). The size of the nonglycosylated band was either 21 kDa (termed type 1, sCJD case BN141) or 19 kDa (termed type 2, sCJD case BN144). Diglycosylated PrP^res^ predominated in the BSE inoculum and in all macaques showing neurologic signs (macaque S4 is shown as a representative example for all symptomatic cases), and the pattern is termed type 2B to distinguish it from type 2 cases in which the monoglycosylated form predominated (Type 2A, sCJD case BN144). Two different mAbs were used for routine immunodetection: 6H4 (left panel) and 3F4 (right panel). Blots were co-stained with a polyclonal anti-actin antiserum. BSE, bovine spongiform encephalopathy; sCJD, sporadic Creutzfeldt-Jakob disease; PK, proteinase K; mAb, monoclonal antibody.

**Figure 4:**
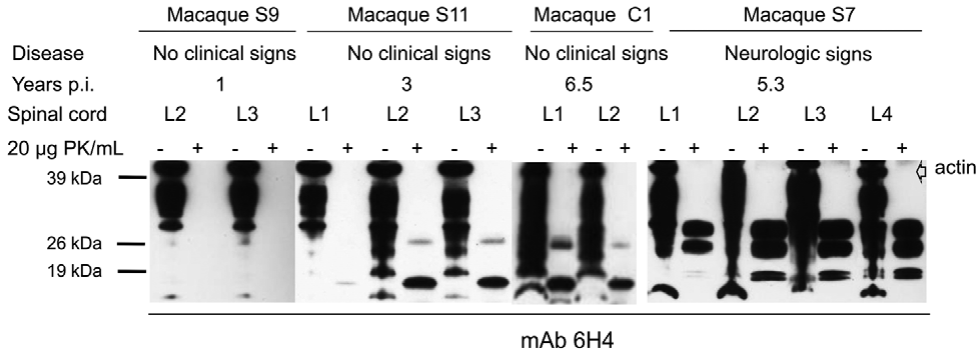
Western blot analysis of bovine spongiform encephalopathy proteinease-resistant prion protein (PrP^res^) (lumbar spinal cord segments) in preclinical and clinically ill macaques. An atypical PrP^res^ pattern was detectable in macaques euthanized during incubation from 3 years p.i. (macaque S11) to 6.5 years p.i. (macaque C1). All samples were co-stained with an anti-actin-antiserum. p.i., postinoculation; PK, proteinase K; mAb, monoclonal antibody.

**Figure 5:**
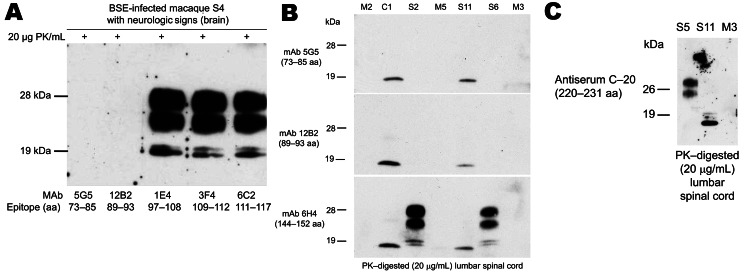
A) Epitope mapping of proteinease-resistant prion protein (PrP^res^) by Western immunblot analyses (thalamus) from a macaque showing neurologic signs. The PK-sensitive N terminal fragment (mAb 5G5) and the adjacent region showing a variable PK sensitivity (mAb 12B2) were completely digested by the proteinease. MAbs 1E4, 3F4, and 6C2 detected the PrP^res^ triplet termed type 2B signature. B) Epitope mapping of PrP^res^ by Western blot analyses (lumbar spinal cord segment L_2_) in clinically ill (S2, S6), subclinical (C1), and preclinical (S11) macaques. In preclinical macaques, mAbs 5G5, 12B2, and 6H4 detected a 17 kDa PrP^res^ fragment. Tissue samples from mock (M) controls were completely negative for PrP^res^. C) Epitope mapping of PrP^res^ by Western blot analyses (lumbar spinal cord segment L_2_) in a clinically ill macaque (S5), a preclinical macaque (S11), and a non–BSE-infected age-/sex-matched control macaque (M3). PK-treated tissue homogenates from preclinical macaques could also be immunostained with antiserum C20 directed against the C-terminus of the atypical 17-kDa fragment. BSE, bovine spongiform encephalopathy; PK, proteinase K; mAb, monoclonal antibody; M2, M3, M5, noninfected macaques.

Among single-dosed macaques, gait ataxia developed in S1–S8, and CSF samples were positive for 14–3-3p from 3.7 through 5.3 years postinoculation; no differences in incubation periods were noted for macaques that received 5 g or 16 g of BSE inoculum (Table 2, Figure 2). Postmortem examinations of all macaques showing neurologic signs detected a type 2B PrP^res^ signature in different brain areas (obex region, cerebellum/deep nuclei, pons, thalamus, caudate nucleus, cortex cerebri) and in all spinal cord segments examined (C_1_–L_4_) (Table 2, Figures 3, 4). The type 1–specific monoclonal antibody 12B2 could not detect any PrP^res^ triplets in these specimens (Figure 5, panel A). Typical spongiform changes were seen in hemotoxylin and eosin–stained brain tissue sections (data not shown).

**Figure 6:**
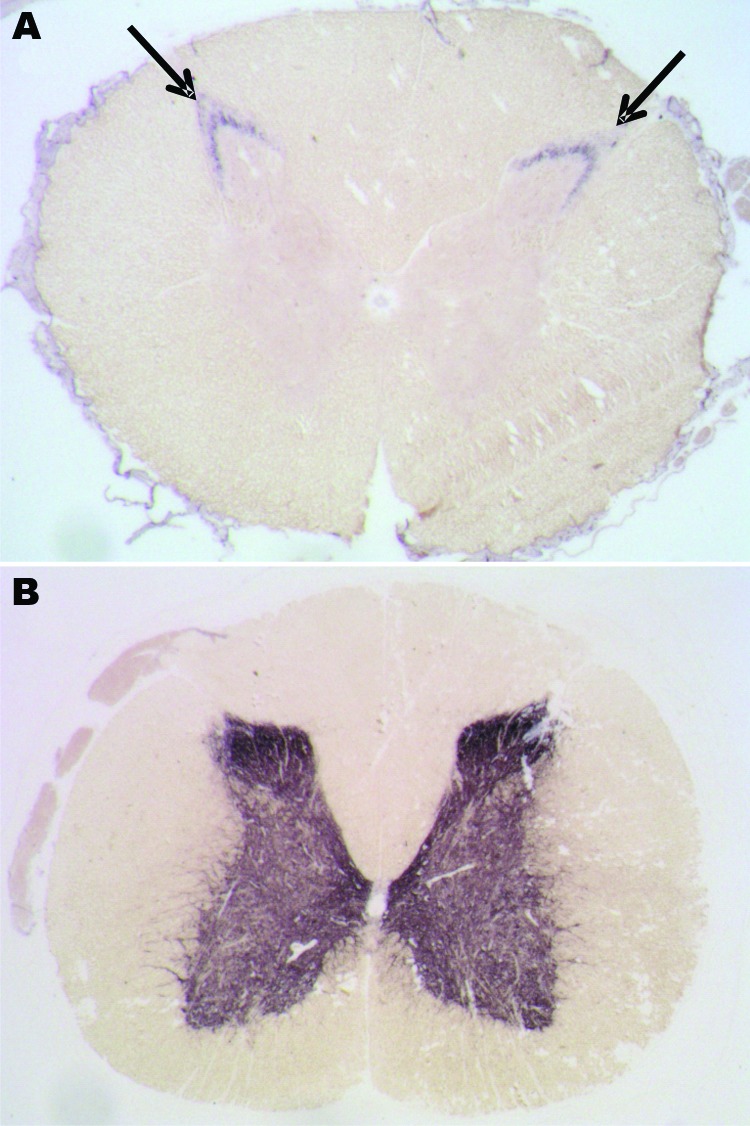
Paraffin-embedded tissue blot analyses of lumbar spinal cord segments from the preclinical macaque S14 (A) and a clinically ill macaque (B) for detection and localization of proteinease-resistant prion protein (PrP^res)^ ) deposits. These deposits could be detected in the substantia gelatinosa (arrows) of preclinical cases.

Among macaques that received 8–16 g of BSE inoculum (C1–C3), no behavioral changes, gait ataxia, or 14–3-3p-positive CSF were detected. However, as they got older, obesity and chronically elevated blood glucose concentrations (>126 mg/dL) developed and were followed by a rapid decrease in body weight. For humane reasons, these animals were euthanized at 6.5 years (C1, C2) and 8.8 years (C3) postinoculation. Retrospective analyses of plasma samples detected normal insulin levels at 3–4 years of age, followed by hyperinsulinemia and a progressive decline in plasma insulin levels. Postmortem examinations of pancreatic tissue sections indicated replacement of the normal islet architecture by islet-associated amyloid and marked reduction of α- and β-cell mass (data not shown). On the basis of the typical changes during the prediabetic phase, clinical parameters, and pancreas histopathology, type 2 diabetes (a common disease for nonhuman primates in captivity) was diagnosed. We detected neither spongiform changes nor PrP^res^ deposits in the brains of these 3 macaques (Table 2). However, an atypical PrP^res^ pattern, a 26-kDa fragment, and a 17-kDa fragment were found in lumbar, but not other, spinal cord segments in 2 of the 3 macaques (Table 2, Figure 4). Paraffin-embedded tissue blot analyses confirmed Western immunoblot results (Figure 6). Paraffin-embedded tissue blot analyses of lymphoid tissues (GALT, lymph nodes) revealed PrP^res^ deposits in 3 macaques (Table 2).

From 3 years postinoculation onward, we also detected an atypical PrP^res^ pattern in lumbar spinal cord segments in 4 of 6 macaques that had received a single 5-g dose of BSE inoculum (macaques S11, S12, S14, S15; Table 2, Figure 4). The lumbar part of the spinal cord is probably the primary site of prion entry into the simian CNS after oral uptake of the BSE agent (data not shown). We did not detect histopathologic changes in hemotoxylin and eosin–stained brain tissue sections from macaques before clinical signs developed (S9–S15, C1–C3).

The atypical molecular signature found in lumbar segments of macaques with subclinical infection was characterized by the predominance of a PrP^res^ fragment, which migrated at the 17-kDa region of the sodium dodecyl sulfate polyacrylamide gel. A second PrP^res^ molecule migrated at the area of 26 kDa but was sometimes hardly visible (Figure 4). Epitope mapping was conducted to characterize these 2 PrP molecules. Paradoxically, all antibodies, including monoclonal antibody 12B2 (Figure 5, panel B), and polyclonal antiserum recognizing the C-terminus (data not shown) could bind to both fragments. Non-PrP antibodies did not bind to the atypical fragments (data not shown). Deglycosylation of PK–treated lumbar spinal cord samples by using peptide -N-glycosidase F treatment resulted in a single band with a molecular weight of ≈22–23 kDa in animals with preclinical/subclinical infection compared with a single 19–20 kDa band in macaques showing neurologic signs (data not shown).

**Figure 7:**
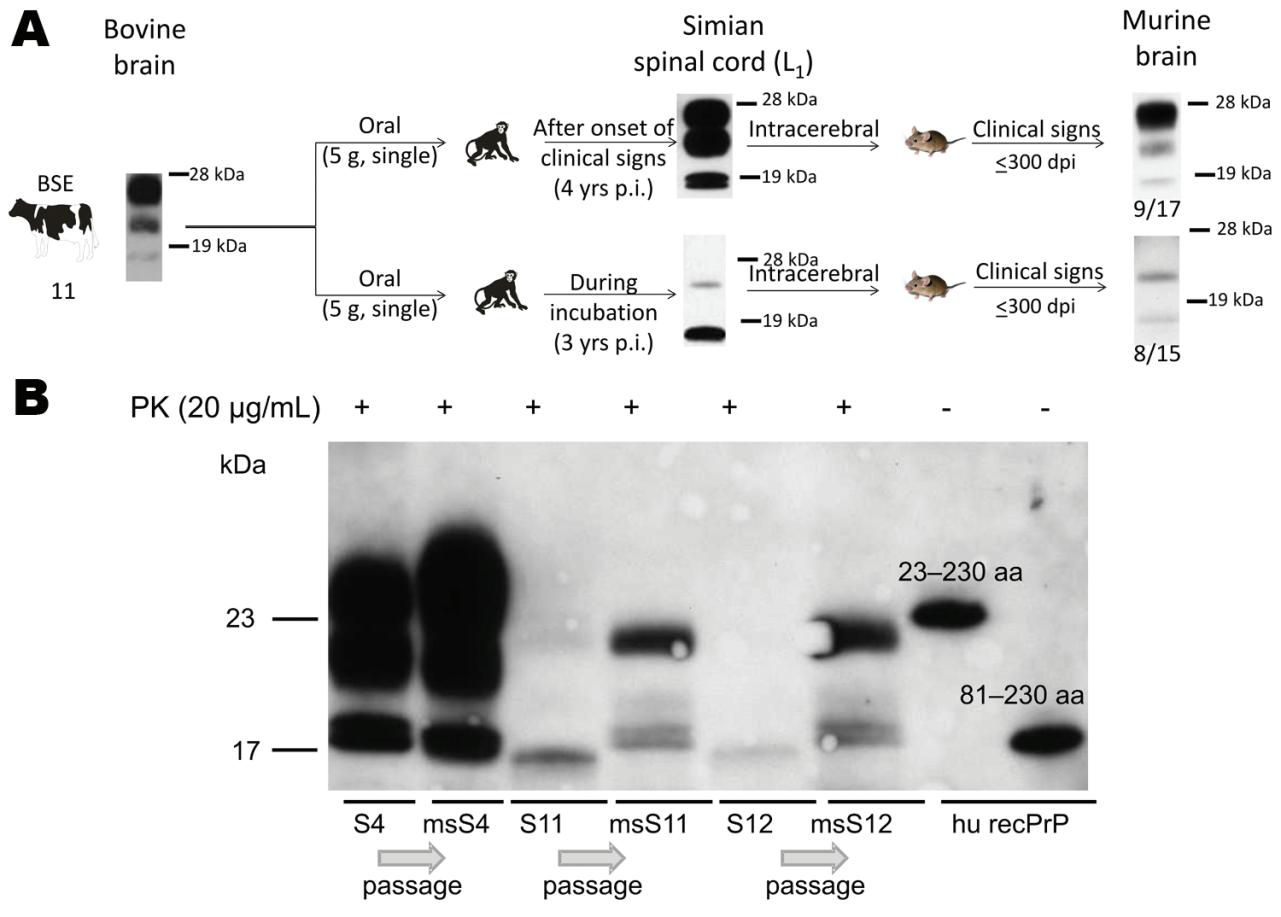
A) Summary of studies of BSE transmission to macaques and subsequent passage of lumbar spinal cord tissues (L_2_–L_3_) from a symptomatic and a preclinical macaque to BoTg110 mice. Inoculation of typical type 2B and atypical material from macaques to transgenic mice caused disease in 9 (53%) of 17 mice and in 8 (53%) of 15 mice, respectively, with no significant differences in incubation periods. B). Western immunoblots of PK-treated lumbar spinal cord samples from BSE-infected macaques (S4, S11, S12) and the corresponding bioassay results (msS4, msS11, msS12) in BoTg110 mice inoculated with simian lumbar spinal cord tissue homogenates. Analyses of the PrP^res^, proteinease-resistant prion protein; (PrP^res^) profile by Western immunoblot using mAb 6H4 showed a conserved BSE glycoform signature after the first passage when typical type 2B material collected from a macaque showing neurologic signs was inoculated into BoTg110 mice. However, the atypical PrP^res^ pattern detected in preclinical cases was not stable after the first passage into BoTg110 mice. Recombinant PrP fragments (23–230 aa and 81–230 aa) were included to characterize molecular weights of the atypical fragments. BSE, bovine spongiform encephalopathy; p.i., postinoculation; dpi, days postinoculation; PK, proteinase K; hu recPrP, human recombinant PrP.

The reason for the abnormal migration behavior of the atypical fragments remains to be determined. When lumbar spinal cord tissue homogenates were intracerebrally inoculated into mice transgenic for the bovine *PRNP* gene, samples from symptomatic (type 2B signature) and asymptomatic macaques (abnormal signature) were infectious and caused disease in mice (53% diseased mice in both groups) with no statistically significant difference in the incubation periods (355 ± 41 vs. 372 ± 7 days postinoculation, respectively, *P*_logrank_ test not significant) (Figure 7). The percentage of mice inoculated with type 2B or non–type 2B material that showed clinical signs was low, probably because of the small amount of gray matter (PrP^res^) in the inoculum; attack rates were 100% among mice inoculated with gray matter from brain samples (data not shown). Among mice infected with tissue samples from the asymptomatic animals, a molecular signature that differed from that of the inoculum developed, whereas the type 2B signature found in macaques showing neurologic signs was stable after transmission to mice (Figure 7).

## Discussion

Because only a few macaques have died, it will take longer than previously estimated before all data from low-dose (0.05 and 0.005 g) exposures will be available (data not shown). Although all macaques were originally to be inoculated with BSE-infected cattle brain material on only 1 occasion, 3 of 6 macaques receiving >5 g of inoculum had to be fed on multiple occasions. 

The attack rate after dietary exposure to ≥5 g BSE brain material in 5-year-old adult 129-MM cynomolgus monkeys was 100% (18/18). PrP^res^ deposits could be detected outside the GALT in gut-draining lymph nodes from at least 1 year postinoculation onward. These data show that the dose at which 50% of these nonhuman primates will be infected will be distinctly lower than previously estimated (*4*).

For single-dosed animals, the incubation period was 4.6 years (median, range 3.7–5.3 years). There was no difference between those that received 5 g and 16 g, indicating that <5 g represented the dose at which disease developed in 100% of macaques (LD_100_). The shortest incubation period was detected in macaque S6, which was given 16 g of inoculum on 1 occasion. This short incubation period might have resulted from the extremely high dose. However, retrospective analyses revealed that macaque S6 had a highly stimulated immune system (data not shown), which might also explain the short incubation period. The low variability of incubation periods (4.3–5.3 years, excluding 3.7 years for macaque S6) was probably the result of the high dose. When we inoculated macaques with lower doses (data not shown) or when macaques were inoculated on multiple occasions, incubation periods were highly variable. After the macaques received multiple oral doses, clinical signs of a spongiform disease had not developed by 6.5 (2 of 3 macaques) to 8.8 years (1 of 3 macaques) postinoculation although they received an LD_100_ on day 1 (Table 1).

Unfortunately, type 2 diabetes developed in all 3 of these macaques as they aged, and they had to be euthanized for humane reasons at the indicated time points. At postmortem examinations, lumbar spinal cord segments were PrP^res^ positive for the 2 macaques (C1 and C2) euthanized 6.5 years postinoculation. We estimate that incubation periods in these 2 animals must be at least 7 years because it took ≥6 months until PrP^res^ deposits were also detectable in the cerebellum/cortex cerebri, thereby causing clinical signs (data not shown). In the third macaque (C3) euthanized 8.8 years postinoculation with a cumulative dose of 16 g, PrP^res^ deposits could only be detected outside the CNS, thereby indicating an estimated incubation period >10 years. Similar results have been described for hamsters orally infected with the scrapie strain 263K on a single or multiple occasions. In that study, a cumulative dose significantly prolonged incubation periods, although hamsters were given much lower doses than were the macaques (*13*). The upper reference margin using 3× the standard deviation (3σ) of animal group I is 1.52 years, corresponding to a calculated upper limit of the incubation period of 6.1 years after a single high-dose exposure (5–16 g each). This calculated incubation period is distinctly lower than the estimated incubation time of >7–10 years within animals of group III, indicating a biological effect of the successive BSE challenge mode on the incubation time in the macaque model.

The discrepancy between the low number of vCJD cases in the United Kingdom to date and the higher prevalence of infected humans estimated on the basis of retrospective biopsy analyses (*27*,*28*) indicates the existence of pre- or subclinical cases, perhaps as a result of a low-dose exposure to BSE-contaminated material or a less susceptible *PRNP* genotype. We showed that multiple exposures to high doses of BSE prolonged incubation periods in a nonhuman primate model. These findings show that a successive BSE challenge mode might contribute to the development of pre- or subclinical cases despite a susceptible *PRNP* phenotype and an LD_100_. This finding is relevant because it is quite likely that most of the UK population has been exposed to BSE-contaminated food on multiple occasions (*5*,*6*).

The underlying mechanism of a prolonged incubation period after multiple exposures to an agent that induces a transmissible spongiform encephalopathy is not known (*6*,*13*). Theoretically, interference between types or strains could have caused this phenomenon, as has been shown by others (*14*,*19*,*29*,*30*). However, Diringer et al. (*13*) used 1 well-defined laboratory scrapie strain (263K) that could also cause prolonged incubation periods in Syrian hamsters after multiple oral exposures. Their finding shows that at least 1 other not-yet identified mechanism causes prolonged incubation periods after multiple oral exposures to agents that induce transmissible spongiform encephalopathy.

Unexpectedly, we detected a non–type 2B PrP^res^ pattern in preclinical cases from 3 years postinoculation onward. Transmission studies in BoTg110 mice showed that tissues were infectious but that this atypical molecular signature was not stable after the first passage to transgenic mice carrying the bovine PrP gene (Figure 7). However, the PK-sensitive N terminal part, the variable region of PK, and the C-terminal end were detectable in both atypical PrP molecules by epitope mapping studies. Thus, at least the 17-kDa molecule showed migration behavior on sodium dodecyl sulfate polyacrylamide gel electrophoresis, which did not correlate with its formal molecular weight. Posttranslational modifications can cause a gel-shifting phenomenon (i.e., anomalous gel mobility), as observed for the phosphorylated tau protein (*31*). However, it remains to be determined which mechanism caused this anomalous gel mobility. This atypical signature probably reflects neither types nor strains but rather an intermediate conformation of the pathologic PrP.

In conclusion, the LD_100_ of brain from BSE-infected cattle for 129-MM 5-year-old adult macaques exposed on 1 occasion is <5 g. However, this dose did not cause disease within a prolonged incubation time when animals were exposed on multiple occasions. This finding may be relevant for modeling exposure risks for foodborne prion diseases including chronic wasting disease (*32*). Moreover, the time-dependent shift of the molecular signature might be relevant for retrospective analyses of biopsy samples most likely from animals with pre- or subclinical vCJD.
